# {*N*,*N*-Bis[bis­(2,2,2-tri­fluoro­eth­oxy)phosphan­yl]methyl­amine-κ^2^ *P*,*P*′}bis­(η^5^-cyclo­penta­dien­yl)titanium(II)

**DOI:** 10.1107/S1600536813014244

**Published:** 2013-05-31

**Authors:** Martin Haehnel, Sven Hansen, Anke Spannenberg, Torsten Beweries

**Affiliations:** a Leibniz-Institut für Katalyse e. V. an der Universität Rostock, Albert-Einstein-Strasse 29a, 18059 Rostock, Germany

## Abstract

The title compound, [Ti(C_5_H_5_)_2_(C_9_H_11_F_12_NO_4_P_2_)], is a four-membered titanacycle obtained from the reaction of Cp_2_Ti(η^2^-Me_3_SiC_2_SiMe_3_) and CH_3_N[P(OCH_2_CF_3_)_2_]_2_ {*N*,*N*-bis­[bis­(tri­fluoro­eth­oxy)phosphan­yl]methyl­amine, tfepma}. The Ti^II^ atom is coordinated by two cyclo­penta­dienyl (Cp) ligands and the chelating tfepma ligand in a strongly distorted tetra­hedral geometry. The mol­ecule is located on a mirror plane.

## Related literature

For other titanocene complexes with four-membered metallacycles [TiPNP], see: Haehnel *et al.* (2012 ▶). For selected examples of four-membered metallacycles with a chelating tfepma ligand, see: *M* = Rh, Esswein *et al.* (2005 ▶, 2007 ▶); *M* = Ir, Heyduk & Nocera (1999 ▶, 2000 ▶); Gray *et al.* (2004 ▶); Veige *et al.* (2005 ▶); Esswein *et al.* (2008 ▶). The starting alkyne complex Cp_2_Ti(η^2^-Me_3_SiC_2_SiMe_3_) is described by Burlakov *et al.* (1988 ▶).
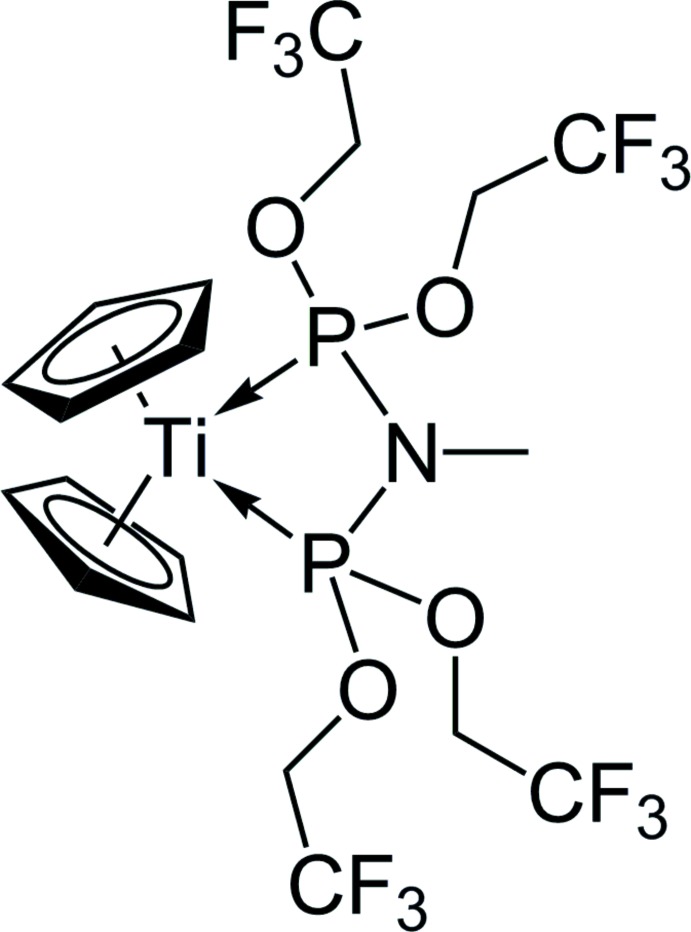


## Experimental

### 

#### Crystal data

[Ti(C_5_H_5_)_2_(C_9_H_11_F_12_NO_4_P_2_)]
*M* *_r_* = 665.21
Orthorhombic, 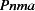

*a* = 14.6494 (2) Å
*b* = 20.0535 (3) Å
*c* = 8.7694 (1) Å
*V* = 2576.20 (6) Å^3^
*Z* = 4
Mo *K*α radiation
μ = 0.57 mm^−1^
*T* = 150 K
0.42 × 0.41 × 0.16 mm

#### Data collection

Bruker Kappa APEXII DUO diffractometer
Absorption correction: multi-scan (*SADABS*; Bruker, 2008 ▶) *T*_min_ = 0.90, *T*_max_ = 1.00
60903 measured reflections
3420 independent reflections
2988 reflections with *I* > 2σ(*I*)
*R*_int_ = 0.033

#### Refinement

*R*[*F* ^2^ > 2σ(*F* ^2^)] = 0.031
*wR*(*F* ^2^) = 0.079
*S* = 1.06
3420 reflections
191 parameters
H atoms treated by a mixture of independent and constrained refinement
Δρ_max_ = 0.51 e Å^−3^
Δρ_min_ = −0.40 e Å^−3^

### 

Data collection: *APEX2* (Bruker, 2011 ▶); cell refinement: *SAINT* (Bruker, 2009 ▶); data reduction: *SAINT*; program(s) used to solve structure: *SHELXS97* (Sheldrick, 2008 ▶); program(s) used to refine structure: *SHELXL97* (Sheldrick, 2008 ▶); molecular graphics: *XP* in *SHELXTL* (Sheldrick, 2008 ▶); software used to prepare material for publication: *SHELXTL*.

## Supplementary Material

Crystal structure: contains datablock(s) I, global. DOI: 10.1107/S1600536813014244/bt6910sup1.cif

Structure factors: contains datablock(s) I. DOI: 10.1107/S1600536813014244/bt6910Isup2.hkl

Additional supplementary materials:  crystallographic information; 3D view; checkCIF report

## Figures and Tables

**Fig. 1.**
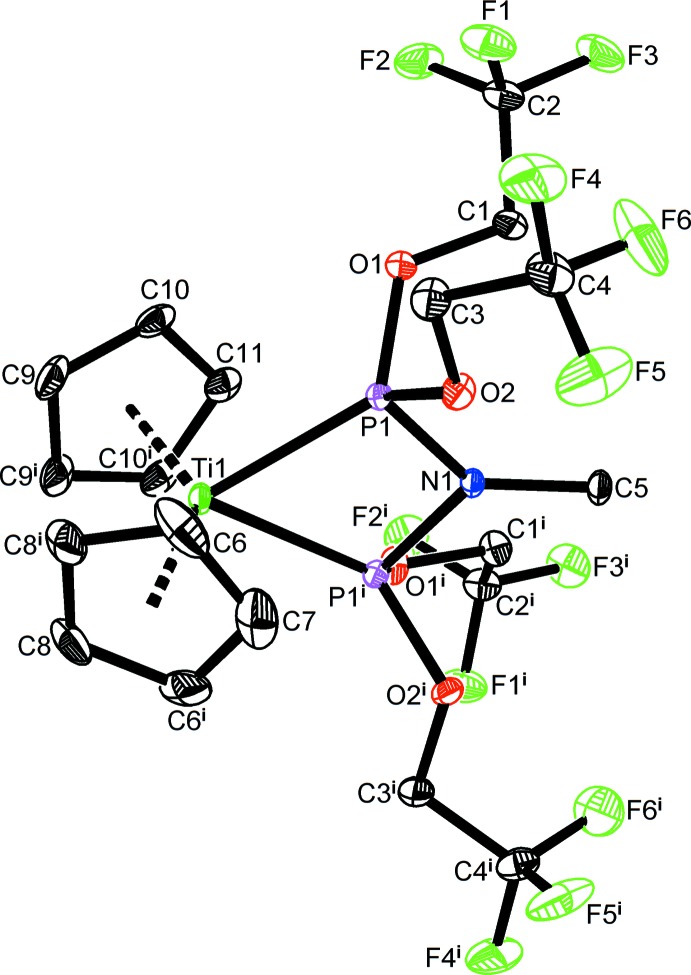
The molecular structure of the title compound in the crystal (symmetry code to generate equivalent atoms: (i) x, -y + 1/2, z). Hydrogen atoms are omitted for clarity. Displacement ellipsoids are drawn at the 30% probability level.

## supplementary crystallographic information

### Comment

The reaction of tfepma with the titanocene precursor Cp_2_Ti(*η*^2^-Me_3_SiC_2_SiMe_3_) was investigated to synthesize a new 4-membered hetero-metallacycle. In this reaction, the spectator ligand Me_3_SiC_2_SiMe_3_ is replaced by the chelating tfepma ligand, which is binding over both phosphorus atoms to result in the four membered metallacycle.

In the title compound the titanium center is coordinated by two Cp units and the chelating tfepma ligand (Fig. 1). The molecule is located on a mirror plane which passes through H5A, C5, N1, Ti1, C7, H7, C11 and H11. The geometry at the titanium center is found to be strongly distorted tetrahedral. The largest deviation from the ideal tetrahedral angle is observed for P1—Ti1—P1^i^ [63.94 (2)°, symmetry code to generate equivalent atoms: (i) *x*, -*y* + 1/2, *z*]. The four membered metallacycle Ti1,P1,N1,P1^i^ is planar with a mean deviation from the best plane of 0.015 Å. The following bond lengths and angles of the cyclic unit were observed: Ti1—P1 = 2.4266 (4) Å, P1—N1 = 1.696 (1) Å, P1—N1—P1^i^ = 98.51 (8)°.

### Experimental

To a stirred solution of Cp_2_Ti(η^2^-Me_3_SiC_2_SiMe_3_) (150 mg, 0.430 mmol) in 5 ml of toluene was added a solution of tfepma (210 mg, 0.430 mmol) in 5 ml of toluene at room temperature. The colour of the reaction mixture instantly changed from brown to dark green. After additional stirring for 1 h, all volatiles were removed in vacuum and the resulting green precipitate was dissolved in 7 ml of toluene and stored at -30°C for several weeks. The resulting dark green single crystals were filtered, washed with cold *n*-hexane and dried in vacuum. Yield: 96% (274 mg, 0.412 mmol).

### Refinement

H5A and H5B were found from the difference Fourier map and refined freely. All other H atoms were placed in idealized positions with d(C—H) = 0.95 Å (CH), 0.99 Å (CH_2_) and refined using a riding model with *U*_iso_(H) fixed at 1.2 *U*_eq_(C).

### Crystal data

**Table:**

[Ti(C_5_H_5_)_2_(C_9_H_11_F_12_NO_4_P_2_)]	*D*_x_ = 1.715 Mg m^−^^3^
*M**_r_* = 665.21	Mo *K*α radiation, λ = 0.71073 Å
Orthorhombic, *P**n**m**a*	Cell parameters from 9979 reflections
*a* = 14.6494 (2) Å	θ = 2.5–28.6°
*b* = 20.0535 (3) Å	µ = 0.57 mm^−^^1^
*c* = 8.7694 (1) Å	*T* = 150 K
*V* = 2576.20 (6) Å^3^	Prism, dark green
*Z* = 4	0.42 × 0.41 × 0.16 mm
*F*(000) = 1336	

### Data collection

**Table:**

Bruker Kappa APEXII DUO diffractometer	3420 independent reflections
Radiation source: fine-focus sealed tube	2988 reflections with *I* > 2σ(*I*)
Curved graphite monochromator	*R*_int_ = 0.033
Detector resolution: 8.3333 pixels mm^-1^	θ_max_ = 28.7°, θ_min_ = 2.5°
ω and phi scans	*h* = −19→19
Absorption correction: multi-scan (*SADABS*; Bruker, 2008)	*k* = −27→24
*T*_min_ = 0.90, *T*_max_ = 1.00	*l* = −11→11
60903 measured reflections	

### Refinement

**Table:**

Refinement on *F*^2^	Primary atom site location: structure-invariant direct methods
Least-squares matrix: full	Secondary atom site location: difference Fourier map
*R*[*F*^2^ > 2σ(*F*^2^)] = 0.031	Hydrogen site location: inferred from neighbouring sites
*wR*(*F*^2^) = 0.079	H atoms treated by a mixture of independent and constrained refinement
*S* = 1.06	*w* = 1/[σ^2^(*F*_o_^2^) + (0.0339*P*)^2^ + 1.9223*P*] where *P* = (*F*_o_^2^ + 2*F*_c_^2^)/3
3420 reflections	(Δ/σ)_max_ = 0.001
191 parameters	Δρ_max_ = 0.51 e Å^−^^3^
0 restraints	Δρ_min_ = −0.40 e Å^−^^3^

### Special details

**Table:**

Geometry. All e.s.d.'s (except the e.s.d. in the dihedral angle between two l.s. planes) are estimated using the full covariance matrix. The cell e.s.d.'s are taken into account individually in the estimation of e.s.d.'s in distances, angles and torsion angles; correlations between e.s.d.'s in cell parameters are only used when they are defined by crystal symmetry. An approximate (isotropic) treatment of cell e.s.d.'s is used for estimating e.s.d.'s involving l.s. planes.
Refinement. Refinement of *F*^2^ against ALL reflections. The weighted *R*-factor *wR* and goodness of fit *S* are based on *F*^2^, conventional *R*-factors *R* are based on *F*, with *F* set to zero for negative *F*^2^. The threshold expression of *F*^2^ > σ(*F*^2^) is used only for calculating *R*-factors(gt) *etc*. and is not relevant to the choice of reflections for refinement. *R*-factors based on *F*^2^ are statistically about twice as large as those based on *F*, and *R*- factors based on ALL data will be even larger.

### Fractional atomic coordinates and isotropic or equivalent isotropic displacement parameters (Å^2^)

**Table:**

	*x*	*y*	*z*	*U*_iso_*/*U*_eq_	
C1	0.92098 (11)	0.13133 (8)	0.72113 (17)	0.0261 (3)	
H1A	0.9235	0.1703	0.6520	0.031*	
H1B	0.8574	0.1259	0.7574	0.031*	
C2	0.95209 (12)	0.06964 (8)	0.63952 (18)	0.0296 (3)	
C3	0.88445 (11)	0.07502 (8)	1.1082 (2)	0.0310 (4)	
H3A	0.9098	0.0719	1.2126	0.037*	
H3B	0.9278	0.0537	1.0367	0.037*	
C4	0.79412 (13)	0.04136 (9)	1.1005 (2)	0.0379 (4)	
C5	0.79887 (14)	0.2500	0.8632 (3)	0.0228 (4)	
H5A	0.8034 (17)	0.2500	0.752 (3)	0.023 (6)*	
H5B	0.7653 (14)	0.2887 (10)	0.897 (2)	0.033 (5)*	
C6	1.01942 (18)	0.19336 (12)	1.3387 (2)	0.0528 (6)	
H6	0.9998	0.1483	1.3320	0.063*	
C7	0.9650 (2)	0.2500	1.3137 (3)	0.0561 (9)	
H7	0.9025	0.2500	1.2850	0.067*	
C8	1.10686 (16)	0.28522 (10)	1.3748 (2)	0.0437 (5)	
H8	1.1578	0.3131	1.3959	0.052*	
C9	1.22452 (11)	0.21453 (9)	1.0707 (3)	0.0404 (5)	
H9	1.2574	0.1868	1.1392	0.049*	
C10	1.16817 (10)	0.19273 (8)	0.9531 (2)	0.0336 (4)	
H10	1.1565	0.1476	0.9265	0.040*	
C11	1.13093 (15)	0.2500	0.8793 (3)	0.0292 (5)	
H11	1.0891	0.2500	0.7964	0.035*	
F1	0.94470 (9)	0.01516 (5)	0.72635 (13)	0.0447 (3)	
F2	1.03855 (8)	0.07332 (6)	0.59488 (13)	0.0441 (3)	
F3	0.90112 (9)	0.05987 (6)	0.51500 (12)	0.0455 (3)	
F4	0.80238 (10)	−0.02315 (5)	1.13574 (17)	0.0569 (4)	
F5	0.73432 (10)	0.06669 (6)	1.1954 (2)	0.0734 (5)	
F6	0.75798 (12)	0.04567 (8)	0.96213 (19)	0.0813 (5)	
N1	0.89119 (11)	0.2500	0.93043 (19)	0.0167 (3)	
O1	0.98087 (7)	0.14063 (5)	0.84642 (12)	0.0253 (2)	
O2	0.87059 (7)	0.14299 (5)	1.06745 (13)	0.0239 (2)	
P1	0.95613 (2)	0.185927 (17)	0.99495 (4)	0.01645 (9)	
Ti1	1.07370 (2)	0.2500	1.12354 (4)	0.01905 (9)	

### Atomic displacement parameters (Å^2^)

**Table:**

	*U*^11^	*U*^22^	*U*^33^	*U*^12^	*U*^13^	*U*^23^
C1	0.0296 (7)	0.0269 (7)	0.0217 (7)	0.0021 (6)	−0.0017 (6)	−0.0068 (6)
C2	0.0402 (9)	0.0244 (7)	0.0242 (7)	−0.0003 (6)	0.0027 (6)	−0.0049 (6)
C3	0.0308 (8)	0.0214 (7)	0.0409 (9)	−0.0025 (6)	−0.0028 (7)	0.0091 (7)
C4	0.0424 (10)	0.0239 (8)	0.0473 (10)	−0.0106 (7)	0.0011 (8)	0.0034 (7)
C5	0.0146 (9)	0.0275 (11)	0.0263 (10)	0.000	−0.0043 (8)	0.000
C6	0.0838 (16)	0.0552 (13)	0.0192 (8)	−0.0323 (12)	−0.0119 (9)	0.0102 (8)
C7	0.0390 (15)	0.113 (3)	0.0162 (11)	0.000	0.0021 (10)	0.000
C8	0.0644 (13)	0.0359 (10)	0.0308 (9)	−0.0074 (9)	−0.0247 (9)	−0.0022 (7)
C9	0.0156 (7)	0.0351 (9)	0.0707 (13)	0.0046 (6)	−0.0038 (8)	0.0044 (9)
C10	0.0183 (7)	0.0251 (8)	0.0573 (11)	0.0015 (6)	0.0114 (7)	−0.0044 (7)
C11	0.0196 (9)	0.0327 (11)	0.0354 (12)	0.000	0.0116 (9)	0.000
F1	0.0710 (8)	0.0228 (5)	0.0403 (6)	−0.0047 (5)	0.0008 (6)	0.0001 (4)
F2	0.0448 (6)	0.0437 (6)	0.0437 (6)	0.0072 (5)	0.0161 (5)	−0.0106 (5)
F3	0.0649 (8)	0.0431 (6)	0.0285 (5)	−0.0021 (6)	−0.0079 (5)	−0.0152 (5)
F4	0.0704 (8)	0.0208 (5)	0.0794 (9)	−0.0123 (5)	0.0114 (7)	0.0060 (6)
F5	0.0586 (8)	0.0395 (7)	0.1222 (13)	−0.0053 (6)	0.0509 (9)	0.0120 (8)
F6	0.0961 (12)	0.0685 (10)	0.0791 (10)	−0.0436 (9)	−0.0491 (9)	0.0132 (8)
N1	0.0136 (7)	0.0184 (7)	0.0180 (7)	0.000	−0.0020 (6)	0.000
O1	0.0226 (5)	0.0269 (5)	0.0263 (5)	0.0036 (4)	−0.0020 (4)	−0.0097 (4)
O2	0.0197 (5)	0.0187 (5)	0.0335 (6)	−0.0029 (4)	0.0012 (4)	0.0048 (4)
P1	0.01472 (15)	0.01642 (16)	0.01822 (16)	−0.00044 (12)	−0.00029 (12)	−0.00094 (12)
Ti1	0.01473 (16)	0.01974 (17)	0.02269 (18)	0.000	−0.00488 (13)	0.000

### Geometric parameters (Å, º)

**Table:**

C1—O1	1.4183 (18)	C8—C8^i^	1.413 (4)
C1—C2	1.500 (2)	C8—Ti1	2.3644 (18)
C1—H1A	0.9900	C8—H8	0.9500
C1—H1B	0.9900	C9—C10	1.392 (3)
C2—F2	1.328 (2)	C9—C9^i^	1.422 (4)
C2—F1	1.3360 (19)	C9—Ti1	2.3670 (17)
C2—F3	1.3374 (19)	C9—H9	0.9500
C3—O2	1.4235 (18)	C10—C11	1.427 (2)
C3—C4	1.487 (2)	C10—Ti1	2.3387 (17)
C3—H3A	0.9900	C10—H10	0.9500
C3—H3B	0.9900	C11—C10^i^	1.427 (2)
C4—F5	1.311 (2)	C11—Ti1	2.300 (2)
C4—F6	1.327 (2)	C11—H11	0.9500
C4—F4	1.336 (2)	N1—P1^i^	1.6959 (11)
C5—N1	1.475 (2)	N1—P1	1.6959 (11)
C5—H5A	0.98 (3)	O1—P1	1.6287 (11)
C5—H5B	0.97 (2)	O2—P1	1.6481 (10)
C6—C8^i^	1.388 (3)	P1—Ti1	2.4266 (4)
C6—C7	1.405 (3)	P1—P1^i^	2.5697 (7)
C6—Ti1	2.3412 (19)	Ti1—C10^i^	2.3387 (17)
C6—H6	0.9500	Ti1—C6^i^	2.3412 (19)
C7—C6^i^	1.405 (3)	Ti1—C8^i^	2.3644 (18)
C7—Ti1	2.305 (3)	Ti1—C9^i^	2.3670 (17)
C7—H7	0.9500	Ti1—P1^i^	2.4266 (4)
C8—C6^i^	1.388 (3)		
			
O1—C1—C2	106.86 (13)	O2—P1—Ti1	129.57 (4)
O1—C1—H1A	110.3	N1—P1—Ti1	98.74 (4)
C2—C1—H1A	110.3	O1—P1—P1^i^	123.89 (4)
O1—C1—H1B	110.3	O2—P1—P1^i^	121.50 (4)
C2—C1—H1B	110.3	Ti1—P1—P1^i^	58.029 (9)
H1A—C1—H1B	108.6	C11—Ti1—C7	157.70 (10)
F2—C2—F1	106.91 (14)	C11—Ti1—C10	35.81 (6)
F2—C2—F3	107.46 (13)	C7—Ti1—C10	150.58 (4)
F1—C2—F3	107.48 (14)	C11—Ti1—C10^i^	35.81 (6)
F2—C2—C1	112.62 (14)	C7—Ti1—C10^i^	150.58 (4)
F1—C2—C1	112.20 (13)	C10—Ti1—C10^i^	58.82 (8)
F3—C2—C1	109.92 (14)	C11—Ti1—C6^i^	150.94 (6)
O2—C3—C4	107.24 (14)	C7—Ti1—C6^i^	35.18 (8)
O2—C3—H3A	110.3	C10—Ti1—C6^i^	162.62 (7)
C4—C3—H3A	110.3	C10^i^—Ti1—C6^i^	118.54 (8)
O2—C3—H3B	110.3	C11—Ti1—C6	150.94 (6)
C4—C3—H3B	110.3	C7—Ti1—C6	35.18 (8)
H3A—C3—H3B	108.5	C10—Ti1—C6	118.54 (8)
F5—C4—F6	106.78 (19)	C10^i^—Ti1—C6	162.61 (7)
F5—C4—F4	106.79 (15)	C6^i^—Ti1—C6	58.05 (12)
F6—C4—F4	108.12 (16)	C11—Ti1—C8^i^	142.46 (8)
F5—C4—C3	112.95 (16)	C7—Ti1—C8^i^	57.85 (9)
F6—C4—C3	111.54 (16)	C10—Ti1—C8^i^	109.11 (7)
F4—C4—C3	110.41 (16)	C10^i^—Ti1—C8^i^	128.38 (8)
N1—C5—H5A	109.7 (15)	C6^i^—Ti1—C8^i^	57.57 (7)
N1—C5—H5B	110.2 (12)	C6—Ti1—C8^i^	34.30 (8)
H5A—C5—H5B	109.9 (14)	C11—Ti1—C8	142.46 (8)
C8^i^—C6—C7	108.0 (2)	C7—Ti1—C8	57.85 (9)
C8^i^—C6—Ti1	73.76 (12)	C10—Ti1—C8	128.38 (8)
C7—C6—Ti1	71.01 (13)	C10^i^—Ti1—C8	109.11 (7)
C8^i^—C6—H6	126.0	C6^i^—Ti1—C8	34.30 (8)
C7—C6—H6	126.0	C6—Ti1—C8	57.57 (7)
Ti1—C6—H6	121.0	C8^i^—Ti1—C8	34.76 (9)
C6^i^—C7—C6	107.9 (3)	C11—Ti1—C9	58.49 (8)
C6^i^—C7—Ti1	73.80 (14)	C7—Ti1—C9	141.84 (8)
C6—C7—Ti1	73.80 (14)	C10—Ti1—C9	34.39 (7)
C6^i^—C7—H7	126.0	C10^i^—Ti1—C9	57.99 (6)
C6—C7—H7	126.0	C6^i^—Ti1—C9	128.36 (8)
Ti1—C7—H7	118.3	C6—Ti1—C9	109.21 (9)
C6^i^—C8—C8^i^	108.03 (13)	C8^i^—Ti1—C9	84.32 (8)
C6^i^—C8—Ti1	71.93 (10)	C8—Ti1—C9	94.62 (8)
C8^i^—C8—Ti1	72.62 (5)	C11—Ti1—C9^i^	58.49 (8)
C6^i^—C8—H8	126.0	C7—Ti1—C9^i^	141.84 (8)
C8^i^—C8—H8	126.0	C10—Ti1—C9^i^	57.99 (6)
Ti1—C8—H8	121.2	C10^i^—Ti1—C9^i^	34.39 (7)
C10—C9—C9^i^	108.31 (10)	C6^i^—Ti1—C9^i^	109.21 (9)
C10—C9—Ti1	71.69 (9)	C6—Ti1—C9^i^	128.36 (8)
C9^i^—C9—Ti1	72.51 (4)	C8^i^—Ti1—C9^i^	94.62 (8)
C10—C9—H9	125.8	C8—Ti1—C9^i^	84.32 (8)
C9^i^—C9—H9	125.8	C9—Ti1—C9^i^	34.97 (9)
Ti1—C9—H9	121.7	C11—Ti1—P1^i^	79.98 (5)
C9—C10—C11	108.07 (16)	C7—Ti1—P1^i^	81.14 (6)
C9—C10—Ti1	73.91 (10)	C10—Ti1—P1^i^	112.52 (5)
C11—C10—Ti1	70.63 (11)	C10^i^—Ti1—P1^i^	82.12 (4)
C9—C10—H10	126.0	C6^i^—Ti1—P1^i^	82.91 (5)
C11—C10—H10	126.0	C6—Ti1—P1^i^	112.97 (7)
Ti1—C10—H10	121.2	C8^i^—Ti1—P1^i^	137.48 (6)
C10—C11—C10^i^	107.2 (2)	C8—Ti1—P1^i^	114.88 (5)
C10—C11—Ti1	73.56 (12)	C9—Ti1—P1^i^	136.94 (6)
C10^i^—C11—Ti1	73.56 (12)	C9^i^—Ti1—P1^i^	114.36 (5)
C10—C11—H11	126.4	C11—Ti1—P1	79.98 (5)
C10^i^—C11—H11	126.4	C7—Ti1—P1	81.14 (6)
Ti1—C11—H11	118.5	C10—Ti1—P1	82.12 (4)
C5—N1—P1^i^	130.36 (4)	C10^i^—Ti1—P1	112.52 (5)
C5—N1—P1	130.36 (4)	C6^i^—Ti1—P1	112.97 (7)
P1^i^—N1—P1	98.51 (8)	C6—Ti1—P1	82.91 (5)
C1—O1—P1	123.73 (9)	C8^i^—Ti1—P1	114.88 (5)
C3—O2—P1	119.25 (10)	C8—Ti1—P1	137.48 (6)
O1—P1—O2	100.73 (6)	C9—Ti1—P1	114.35 (5)
O1—P1—N1	106.29 (7)	C9^i^—Ti1—P1	136.94 (6)
O2—P1—N1	95.62 (6)	P1^i^—Ti1—P1	63.942 (18)
O1—P1—Ti1	120.60 (4)		

Symmetry code: (i) *x*, −*y*+1/2, *z*.
